# 4-[(2*E*)-2-(2-Hy­droxy­benzyl­idene)hydrazin-1-yl]benzonitrile

**DOI:** 10.1107/S1600536812037841

**Published:** 2012-09-08

**Authors:** Shaaban K. Mohamed, Goran A. Bogdanović, Antar A. Abdelhamid, Sladjana B. Novaković, Herman Potgeiter

**Affiliations:** a Manchester Metropolitan University, Chemistry and Environmental Division, Manchester M1 5GD, England; b ’Vinča’ Institute of Nuclear Sciences, Laboratory of Theoretical Physics and Condensed Matter Physics, University of Belgrade, PO Box 522, 11001 Belgrade, Serbia; c School of Research, Enterprise & Innovation, Manchester Metropolitan University, Manchester M1 5GD, England

## Abstract

The asymmetric unit of the title Schiff base, C_14_H_11_N_3_O, contains two independent mol­ecules which have similar conformations. The dihedral angles between the benzene rings are 4.19 (9) and 14.18 (9)° in the two mol­ecules. An intra­molecular O—H⋯N hydrogen bond stabilizes the mol­ecular conformation of each mol­ecules. The crystal packing is dominated by pairs of equivalent N—H⋯N and C—H⋯O hydrogen bonds which arrange the mol­ecules into layers parallel to (-111).

## Related literature

For azomethines, see: Archibald *et al.* (1994 ▶); Harada *et al.* (1999 ▶); Ogawa *et al.* (1998 ▶). For the biological properties of Schiff bases, see: Lozier *et al.* (1975 ▶); Dao *et al.* (2000 ▶). For their coordination chemistry, see: Kargar *et al.* (2009 ▶); Yeap *et al.* (2009 ▶). For the structure of related Schiff bases reported by our group, see: Mohamed, Abdelhamid *et al.* (2012 ▶); Mohamed, Akkurt *et al.* (2012 ▶).
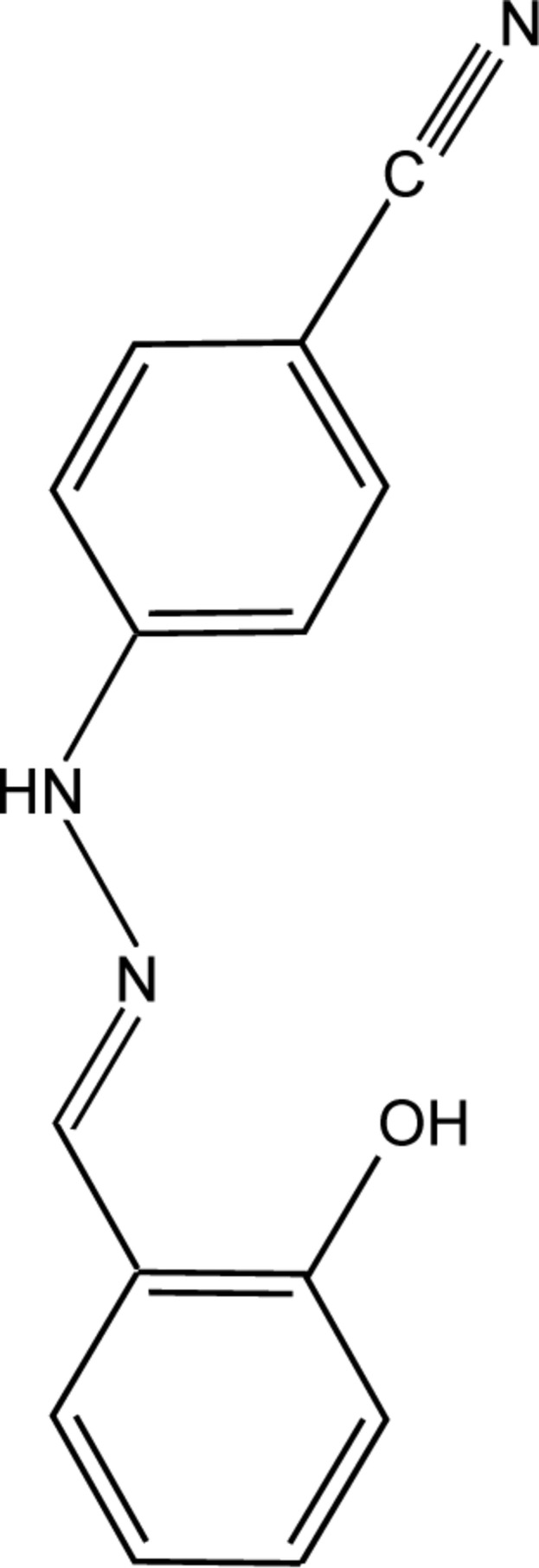


## Experimental

### 

#### Crystal data

C_14_H_11_N_3_O
*M* *_r_* = 237.26
Triclinic, 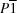

*a* = 8.1917 (7) Å
*b* = 11.6406 (7) Å
*c* = 13.4445 (8) Å
α = 103.006 (5)°
β = 104.387 (6)°
γ = 96.426 (6)°
*V* = 1190.67 (14) Å^3^
*Z* = 4
Cu *K*α radiation
μ = 0.70 mm^−1^
*T* = 293 K
0.26 × 0.15 × 0.14 mm

#### Data collection

Oxford Diffraction Xcalibur (Sapphire3, Gemini) diffractometer
Absorption correction: multi-scan (*CrysAlis PRO*; Oxford Diffraction, 2009 ▶) *T*_min_ = 0.827, *T*_max_ = 1.000
7913 measured reflections
4592 independent reflections
3696 reflections with *I* > 2σ(*I*)
*R*_int_ = 0.020

#### Refinement

*R*[*F* ^2^ > 2σ(*F* ^2^)] = 0.041
*wR*(*F* ^2^) = 0.121
*S* = 1.05
4592 reflections
341 parameters
H atoms treated by a mixture of independent and constrained refinement
Δρ_max_ = 0.16 e Å^−3^
Δρ_min_ = −0.14 e Å^−3^

### 

Data collection: *CrysAlis PRO* (Oxford Diffraction, 2009 ▶); cell refinement: *CrysAlis PRO*; data reduction: *CrysAlis PRO*; program(s) used to solve structure: *SHELXS97* (Sheldrick, 2008 ▶); program(s) used to refine structure: *SHELXL97* (Sheldrick, 2008 ▶); molecular graphics: *ORTEP-3* (Farrugia, 1997 ▶) and *Mercury* (Macrae *et al.*, 2006 ▶)’; software used to prepare material for publication: *WinGX* (Farrugia, 1999 ▶), *PLATON* (Spek, 2009 ▶) and *PARST* (Nardelli, 1995 ▶).

## Supplementary Material

Crystal structure: contains datablock(s) I, global. DOI: 10.1107/S1600536812037841/rz2800sup1.cif

Structure factors: contains datablock(s) I. DOI: 10.1107/S1600536812037841/rz2800Isup2.hkl

Supplementary material file. DOI: 10.1107/S1600536812037841/rz2800Isup3.cml

Additional supplementary materials:  crystallographic information; 3D view; checkCIF report

## Figures and Tables

**Table 1:** Hydrogen-bond geometry (Å, °)

*D*—H⋯*A*	*D*—H	H⋯*A*	*D*⋯*A*	*D*—H⋯*A*
O1*A*—H1*OA*⋯N1*A*	0.98 (2)	1.77 (2)	2.654 (2)	149 (2)
O1*B*—H1*OB*⋯N1*B*	0.90 (2)	1.86 (2)	2.653 (2)	147 (2)
N2*A*—H1*NA*⋯N3*B* ^i^	0.91 (2)	2.17 (2)	3.065 (2)	166 (2)
N2*B*—H1*NB*⋯N3*A* ^ii^	0.89 (2)	2.25 (2)	3.098 (2)	158 (2)
C7*A*—H7*A*⋯O1*B* ^iii^	0.93	2.55	3.427 (2)	156
C7*B*—H7*B*⋯O1*A* ^iv^	0.93	2.49	3.413 (2)	172

Symmetry codes: (i) 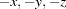; (ii) 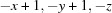; (iii) 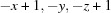; (iv) 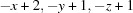.

**Fig. 1.**
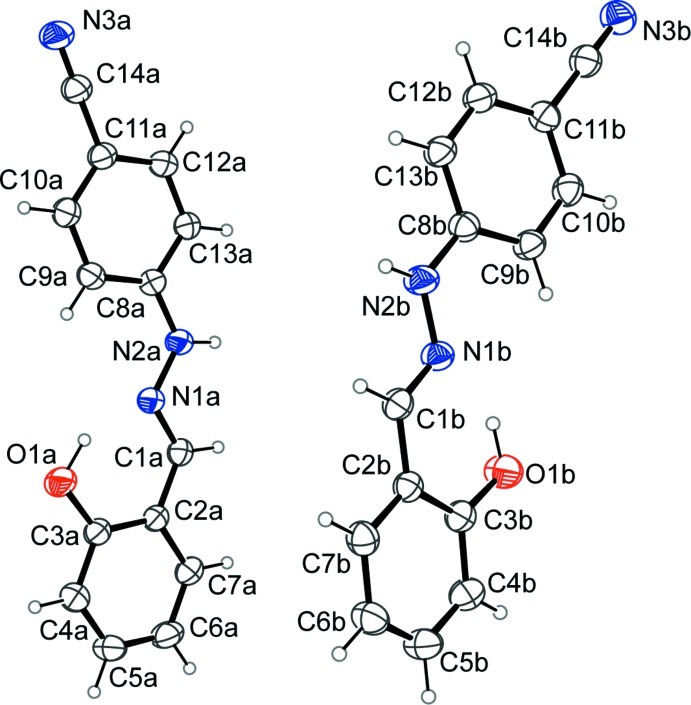
The molecular structure of (I) with atom numbering scheme. Displacement ellipsoids are drawn at the 35% probability level.

**Fig. 2.**
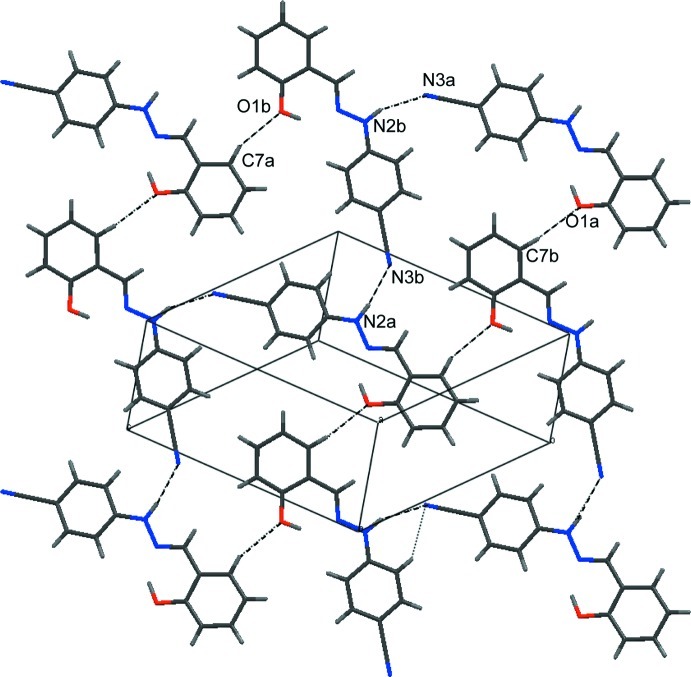
Two dimensional arrangement of A and B molecules via N—H···N and C—H···O hydrogen bonds.

## supplementary crystallographic information

### Comment

Schiff bases have received much attention in recent years (Ogawa *et al.*, 1998; Archibald *et al.*, 1994; Harada *et al.*, 1999) due to their various biological activities and metal chelating properties. In many cases, they were shown to have antibacterial, anticancer, anti- inflammatory and antitoxic properties (Lozier *et al.*, 1975; Dao *et al.*, 2000) and have also been used as versatile ligands in coordination chemistry (Kargar *et al.*, 2009; Yeap *et al.*, 2009). Recently, we reported on the crystal structures of two new Schiff bases (Mohamed, Abdelhamid *et al.*, 2012; Mohamed, Akkurt *et al.*, 2012). As a further investigation of the structures of Schiff base compounds, herin we report the synthesis and crystal structure of the title compound (I) which was obtained unintentionally from the component reaction of cyclohexan-1,3-dione, salicylaldehyde and 4- hydrazinylbenzonitrile in ethanol.

The title compound crystallizes with two independent molecules (A and B) in the asymmetric unit, Fig. 1. The molecules A and B have similar conformation and approximately planar form. In molecules A and B the dihedral angle between the corresponding aromatic rings is 4.19 (9) and 14.18 (9)°, respectively. The torsion angles C8—N2—N1—C1 and N2—N1—C1—C2, within the fragment which connects the rings, are 179.44 (14)/-179.93 (13) and 175.41 (13)/177.44 (12)°, in molecules A and B respectively. All these parameters suggest a somewhat higher planarity of molecule A in comparison to molecule B. The molecules of each type are stabilized by the cyclic intramolecular O1—H1o···N1 hydrogen bond (Table 1). In the crystal packing the A and B molecules mutually interact by the pairs of the strongest N2—H1n···N3 hydrogen bonds (Table 1) which engage the hydrazine donor and the nitirile acceptor from each type of molecule. The chains consisting of A and B molecules further interact by another pair of equivalent C7—H7···O1 interactions to give two dimensional lyres (Fig 2). The interaction between the parallel lyres towards the three-dimensional crystal packing is mostly based on weak van der Waals interactions.

### Experimental

The title compound was prepared unintentionally as a major product from reaction of 112 mg (1 mmol) cyclohexane-1,3-dione, 133 mg (1 mmol) 4-hydrazinylbenzonitrile and 122 mg (1 mmol) salicylaldehyde in 50 ml ethanol. The reaction mixture was refluxed for 5 h. The excess solvent was evaporated under vacuum and the residual resins was triturated with cold acetone. The obtained solid was collected by filtration, dried and washed with acetone. Single crystals suitable for X-ray diffraction were grown from acetone solution of (I) using the slow evaporation method. M. p. 469 K.

### Refinement

H atoms bonded to C atoms were placed at calculated positions, with C—H distances fixed at 0.93 Å and isotropic displacement parameters set equal to 1.2*U*_eq_ of the parent C(*sp*^2^) atoms. H atoms attached to N and O were located in difference Fourier map and refined isotropically.

### Crystal data

**Table:**

C_14_H_11_N_3_O	*Z* = 4
*M**_r_* = 237.26	*F*(000) = 496
Triclinic, *P*1	*D*_x_ = 1.324 Mg m^−^^3^
Hall symbol: -P 1	Cu *K*α radiation, λ = 1.5418 Å
*a* = 8.1917 (7) Å	Cell parameters from 3110 reflections
*b* = 11.6406 (7) Å	θ = 3.5–72.5°
*c* = 13.4445 (8) Å	µ = 0.70 mm^−^^1^
α = 103.006 (5)°	*T* = 293 K
β = 104.387 (6)°	Prismatic, colorless
γ = 96.426 (6)°	0.26 × 0.15 × 0.14 mm
*V* = 1190.67 (14) Å^3^	

### Data collection

**Table:**

Oxford Diffraction Xcalibur (Sapphire3, Gemini) diffractometer	4592 independent reflections
Radiation source: Enhance (Cu) X-ray Source	3696 reflections with *I* > 2σ(*I*)
Graphite monochromator	*R*_int_ = 0.020
Detector resolution: 16.3280 pixels mm^-1^	θ_max_ = 72.6°, θ_min_ = 3.5°
ω scans	*h* = −9→10
Absorption correction: multi-scan (*CrysAlis PRO*; Oxford Diffraction, 2009)	*k* = −14→13
*T*_min_ = 0.827, *T*_max_ = 1.000	*l* = −16→14
7913 measured reflections	

### Refinement

**Table:**

Refinement on *F*^2^	Primary atom site location: structure-invariant direct methods
Least-squares matrix: full	Secondary atom site location: difference Fourier map
*R*[*F*^2^ > 2σ(*F*^2^)] = 0.041	Hydrogen site location: inferred from neighbouring sites
*wR*(*F*^2^) = 0.121	H atoms treated by a mixture of independent and constrained refinement
*S* = 1.05	*w* = 1/[σ^2^(*F*_o_^2^) + (0.0591*P*)^2^ + 0.1257*P*] where *P* = (*F*_o_^2^ + 2*F*_c_^2^)/3
4592 reflections	(Δ/σ)_max_ < 0.001
341 parameters	Δρ_max_ = 0.16 e Å^−^^3^
0 restraints	Δρ_min_ = −0.14 e Å^−^^3^

### Special details

**Table:**

Experimental. Absorption correction: Empirical absorption correction using spherical harmonics, implemented in SCALE3 ABSPACK scaling algorithm. 'CrysAlisPro (Oxford Diffraction, 2009)'

### Fractional atomic coordinates and isotropic or equivalent isotropic displacement parameters (Å^2^)

**Table:**

	*x*	*y*	*z*	*U*_iso_*/*U*_eq_	
O1A	0.48536 (17)	0.59328 (11)	0.66183 (10)	0.0784 (4)	
O1B	0.91964 (17)	−0.08146 (11)	0.25880 (10)	0.0774 (4)	
N1A	0.22126 (15)	0.44483 (11)	0.51588 (9)	0.0526 (3)	
N1B	0.93383 (15)	0.06285 (11)	0.13286 (9)	0.0522 (3)	
N2A	0.09712 (17)	0.40478 (12)	0.42146 (10)	0.0596 (3)	
N2B	0.87286 (17)	0.10497 (12)	0.04621 (10)	0.0602 (3)	
N3A	−0.0385 (2)	0.69952 (14)	0.03913 (11)	0.0808 (5)	
N3B	0.20536 (19)	−0.19989 (13)	−0.37048 (11)	0.0718 (4)	
C1A	0.22866 (18)	0.37735 (12)	0.58006 (11)	0.0515 (3)	
H1A	0.1506	0.3060	0.5593	0.062*	
C1B	1.06978 (18)	0.12410 (12)	0.20323 (11)	0.0497 (3)	
H1B	1.1242	0.1924	0.1917	0.060*	
C2A	0.35367 (18)	0.40831 (12)	0.68322 (11)	0.0480 (3)	
C2B	1.14175 (17)	0.09042 (12)	0.30040 (11)	0.0475 (3)	
C3A	0.47572 (19)	0.51421 (13)	0.72128 (12)	0.0536 (3)	
C3B	1.0654 (2)	−0.00961 (13)	0.32475 (12)	0.0549 (3)	
C4A	0.5895 (2)	0.54087 (15)	0.82180 (13)	0.0662 (4)	
H4A	0.6707	0.6109	0.8464	0.079*	
C4B	1.1394 (2)	−0.03657 (15)	0.41970 (13)	0.0676 (4)	
H4B	1.0883	−0.1025	0.4362	0.081*	
C5A	0.5828 (2)	0.46422 (17)	0.88535 (13)	0.0706 (5)	
H5A	0.6591	0.4832	0.9531	0.085*	
C5B	1.2868 (2)	0.03347 (17)	0.48887 (13)	0.0735 (5)	
H5B	1.3355	0.0142	0.5519	0.088*	
C6A	0.4644 (2)	0.35943 (17)	0.84996 (13)	0.0703 (4)	
H6A	0.4605	0.3079	0.8934	0.084*	
C6B	1.3643 (2)	0.13218 (16)	0.46669 (13)	0.0695 (4)	
H6B	1.4646	0.1793	0.5142	0.083*	
C7A	0.3520 (2)	0.33197 (14)	0.74959 (12)	0.0600 (4)	
H7A	0.2730	0.2608	0.7254	0.072*	
C7B	1.29124 (19)	0.16031 (14)	0.37294 (12)	0.0574 (4)	
H7B	1.3429	0.2272	0.3580	0.069*	
C8A	0.07493 (18)	0.46900 (12)	0.34649 (11)	0.0511 (3)	
C8B	0.73501 (18)	0.04005 (13)	−0.03593 (11)	0.0507 (3)	
C9A	0.1698 (2)	0.58289 (14)	0.36363 (12)	0.0589 (4)	
H9A	0.2530	0.6184	0.4279	0.071*	
C9B	0.66057 (19)	−0.07671 (13)	−0.04234 (12)	0.0549 (3)	
H9B	0.7039	−0.1134	0.0108	0.066*	
C10A	0.1391 (2)	0.64216 (14)	0.28470 (12)	0.0622 (4)	
H10A	0.2015	0.7182	0.2965	0.075*	
C10B	0.5233 (2)	−0.13687 (13)	−0.12740 (12)	0.0571 (4)	
H10B	0.4750	−0.2146	−0.1314	0.068*	
C11A	0.0169 (2)	0.59024 (13)	0.18796 (12)	0.0558 (4)	
C11B	0.45513 (19)	−0.08366 (13)	−0.20774 (11)	0.0539 (3)	
C12A	−0.0775 (2)	0.47666 (14)	0.17121 (12)	0.0587 (4)	
H12A	−0.1604	0.4412	0.1068	0.070*	
C12B	0.5284 (2)	0.03318 (14)	−0.20013 (12)	0.0595 (4)	
H12B	0.4834	0.0704	−0.2526	0.071*	
C13A	−0.0485 (2)	0.41730 (13)	0.24934 (11)	0.0575 (4)	
H13A	−0.1118	0.3416	0.2374	0.069*	
C13B	0.6654 (2)	0.09324 (13)	−0.11637 (12)	0.0582 (4)	
H13B	0.7134	0.1709	−0.1127	0.070*	
C14A	−0.0144 (2)	0.65174 (15)	0.10526 (13)	0.0638 (4)	
C14B	0.3157 (2)	−0.14810 (14)	−0.29808 (13)	0.0587 (4)	
H1NA	0.021 (2)	0.3362 (16)	0.4094 (14)	0.077 (5)*	
H1NB	0.934 (2)	0.1706 (16)	0.0406 (13)	0.072 (5)*	
H1OA	0.397 (3)	0.559 (2)	0.5941 (17)	0.103 (7)*	
H1OB	0.886 (3)	−0.0526 (18)	0.2020 (16)	0.090 (7)*	

### Atomic displacement parameters (Å^2^)

**Table:**

	*U*^11^	*U*^22^	*U*^33^	*U*^12^	*U*^13^	*U*^23^
O1A	0.0813 (8)	0.0629 (7)	0.0783 (8)	−0.0142 (6)	−0.0038 (7)	0.0360 (6)
O1B	0.0827 (8)	0.0657 (7)	0.0713 (7)	−0.0186 (6)	0.0021 (6)	0.0304 (6)
N1A	0.0542 (7)	0.0505 (6)	0.0489 (6)	0.0056 (5)	0.0068 (5)	0.0149 (5)
N1B	0.0551 (7)	0.0543 (7)	0.0490 (6)	0.0068 (5)	0.0130 (5)	0.0201 (5)
N2A	0.0620 (8)	0.0531 (7)	0.0537 (7)	−0.0011 (6)	−0.0009 (6)	0.0186 (6)
N2B	0.0639 (8)	0.0588 (7)	0.0542 (7)	−0.0017 (6)	0.0061 (6)	0.0255 (6)
N3A	0.0952 (11)	0.0772 (10)	0.0642 (8)	−0.0075 (8)	0.0078 (8)	0.0344 (8)
N3B	0.0674 (9)	0.0678 (9)	0.0666 (8)	−0.0049 (7)	0.0042 (7)	0.0149 (7)
C1A	0.0532 (8)	0.0452 (7)	0.0546 (8)	0.0044 (6)	0.0127 (6)	0.0150 (6)
C1B	0.0511 (7)	0.0469 (7)	0.0535 (7)	0.0052 (6)	0.0171 (6)	0.0170 (6)
C2A	0.0508 (7)	0.0456 (7)	0.0499 (7)	0.0109 (6)	0.0147 (6)	0.0154 (6)
C2B	0.0489 (7)	0.0467 (7)	0.0487 (7)	0.0097 (6)	0.0160 (6)	0.0131 (6)
C3A	0.0546 (8)	0.0493 (7)	0.0563 (8)	0.0079 (6)	0.0107 (6)	0.0190 (6)
C3B	0.0598 (8)	0.0494 (8)	0.0547 (8)	0.0055 (6)	0.0142 (7)	0.0165 (6)
C4A	0.0608 (9)	0.0606 (9)	0.0655 (10)	0.0049 (7)	0.0017 (7)	0.0142 (8)
C4B	0.0817 (11)	0.0636 (10)	0.0624 (9)	0.0119 (8)	0.0186 (8)	0.0291 (8)
C5A	0.0726 (11)	0.0796 (11)	0.0534 (9)	0.0201 (9)	0.0019 (8)	0.0198 (8)
C5B	0.0836 (12)	0.0823 (12)	0.0557 (9)	0.0239 (10)	0.0102 (8)	0.0265 (9)
C6A	0.0845 (12)	0.0761 (11)	0.0603 (9)	0.0232 (9)	0.0188 (8)	0.0351 (8)
C6B	0.0619 (9)	0.0756 (11)	0.0581 (9)	0.0098 (8)	0.0015 (7)	0.0103 (8)
C7A	0.0683 (9)	0.0538 (8)	0.0617 (9)	0.0086 (7)	0.0180 (7)	0.0242 (7)
C7B	0.0532 (8)	0.0548 (8)	0.0602 (8)	0.0051 (6)	0.0139 (7)	0.0116 (7)
C8A	0.0539 (8)	0.0500 (7)	0.0495 (7)	0.0119 (6)	0.0120 (6)	0.0150 (6)
C8B	0.0521 (8)	0.0523 (8)	0.0488 (7)	0.0081 (6)	0.0152 (6)	0.0151 (6)
C9A	0.0634 (9)	0.0555 (8)	0.0499 (8)	0.0006 (7)	0.0058 (7)	0.0140 (6)
C9B	0.0594 (8)	0.0542 (8)	0.0547 (8)	0.0100 (6)	0.0156 (7)	0.0226 (6)
C10A	0.0691 (10)	0.0536 (8)	0.0599 (9)	−0.0006 (7)	0.0134 (7)	0.0180 (7)
C10B	0.0604 (9)	0.0495 (8)	0.0625 (9)	0.0053 (6)	0.0192 (7)	0.0173 (7)
C11A	0.0605 (9)	0.0572 (8)	0.0527 (8)	0.0104 (7)	0.0152 (7)	0.0211 (7)
C11B	0.0520 (8)	0.0563 (8)	0.0518 (8)	0.0065 (6)	0.0147 (6)	0.0128 (6)
C12A	0.0597 (9)	0.0593 (9)	0.0510 (8)	0.0072 (7)	0.0049 (7)	0.0160 (7)
C12B	0.0637 (9)	0.0586 (9)	0.0551 (8)	0.0080 (7)	0.0093 (7)	0.0226 (7)
C13A	0.0608 (9)	0.0486 (8)	0.0558 (8)	0.0032 (6)	0.0058 (7)	0.0140 (6)
C13B	0.0629 (9)	0.0515 (8)	0.0578 (8)	0.0025 (7)	0.0094 (7)	0.0218 (7)
C14A	0.0679 (10)	0.0624 (9)	0.0581 (9)	0.0013 (7)	0.0112 (7)	0.0219 (7)
C14B	0.0591 (9)	0.0559 (8)	0.0608 (9)	0.0058 (7)	0.0169 (7)	0.0171 (7)

### Geometric parameters (Å, º)

**Table:**

O1A—C3A	1.3559 (17)	C5B—C6B	1.377 (3)
O1A—H1OA	0.98 (2)	C5B—H5B	0.9300
O1B—C3B	1.3520 (18)	C6A—C7A	1.376 (2)
O1B—H1OB	0.90 (2)	C6A—H6A	0.9300
N1A—C1A	1.2865 (17)	C6B—C7B	1.382 (2)
N1A—N2A	1.3597 (16)	C6B—H6B	0.9300
N1B—C1B	1.2801 (18)	C7A—H7A	0.9300
N1B—N2B	1.3674 (16)	C7B—H7B	0.9300
N2A—C8A	1.3734 (18)	C8A—C13A	1.3943 (19)
N2A—H1NA	0.914 (18)	C8A—C9A	1.399 (2)
N2B—C8B	1.3709 (18)	C8B—C13B	1.397 (2)
N2B—H1NB	0.894 (18)	C8B—C9B	1.4008 (19)
N3A—C14A	1.140 (2)	C9A—C10A	1.379 (2)
N3B—C14B	1.142 (2)	C9A—H9A	0.9300
C1A—C2A	1.4453 (19)	C9B—C10B	1.374 (2)
C1A—H1A	0.9300	C9B—H9B	0.9300
C1B—C2B	1.4517 (19)	C10A—C11A	1.387 (2)
C1B—H1B	0.9300	C10A—H10A	0.9300
C2A—C7A	1.3951 (19)	C10B—C11B	1.393 (2)
C2A—C3A	1.402 (2)	C10B—H10B	0.9300
C2B—C7B	1.3925 (19)	C11A—C12A	1.395 (2)
C2B—C3B	1.4017 (19)	C11A—C14A	1.439 (2)
C3A—C4A	1.383 (2)	C11B—C12B	1.394 (2)
C3B—C4B	1.391 (2)	C11B—C14B	1.432 (2)
C4A—C5A	1.373 (2)	C12A—C13A	1.370 (2)
C4A—H4A	0.9300	C12A—H12A	0.9300
C4B—C5B	1.367 (3)	C12B—C13B	1.363 (2)
C4B—H4B	0.9300	C12B—H12B	0.9300
C5A—C6A	1.378 (3)	C13A—H13A	0.9300
C5A—H5A	0.9300	C13B—H13B	0.9300
			
C3A—O1A—H1OA	106.5 (13)	C7B—C6B—H6B	120.4
C3B—O1B—H1OB	108.4 (13)	C6A—C7A—C2A	121.60 (15)
C1A—N1A—N2A	116.26 (12)	C6A—C7A—H7A	119.2
C1B—N1B—N2B	117.48 (12)	C2A—C7A—H7A	119.2
N1A—N2A—C8A	121.92 (12)	C6B—C7B—C2B	121.27 (15)
N1A—N2A—H1NA	119.3 (11)	C6B—C7B—H7B	119.4
C8A—N2A—H1NA	118.6 (11)	C2B—C7B—H7B	119.4
N1B—N2B—C8B	120.79 (12)	N2A—C8A—C13A	117.82 (13)
N1B—N2B—H1NB	117.9 (11)	N2A—C8A—C9A	123.00 (13)
C8B—N2B—H1NB	120.8 (11)	C13A—C8A—C9A	119.18 (13)
N1A—C1A—C2A	122.47 (13)	N2B—C8B—C13B	118.37 (13)
N1A—C1A—H1A	118.8	N2B—C8B—C9B	122.92 (13)
C2A—C1A—H1A	118.8	C13B—C8B—C9B	118.71 (13)
N1B—C1B—C2B	122.14 (12)	C10A—C9A—C8A	119.59 (14)
N1B—C1B—H1B	118.9	C10A—C9A—H9A	120.2
C2B—C1B—H1B	118.9	C8A—C9A—H9A	120.2
C7A—C2A—C3A	117.93 (13)	C10B—C9B—C8B	119.81 (13)
C7A—C2A—C1A	119.35 (13)	C10B—C9B—H9B	120.1
C3A—C2A—C1A	122.70 (13)	C8B—C9B—H9B	120.1
C7B—C2B—C3B	118.45 (13)	C9A—C10A—C11A	121.09 (14)
C7B—C2B—C1B	119.12 (13)	C9A—C10A—H10A	119.5
C3B—C2B—C1B	122.43 (13)	C11A—C10A—H10A	119.5
O1A—C3A—C4A	118.18 (14)	C9B—C10B—C11B	121.24 (13)
O1A—C3A—C2A	121.55 (13)	C9B—C10B—H10B	119.4
C4A—C3A—C2A	120.27 (14)	C11B—C10B—H10B	119.4
O1B—C3B—C4B	118.20 (14)	C10A—C11A—C12A	119.13 (13)
O1B—C3B—C2B	122.02 (13)	C10A—C11A—C14A	121.12 (14)
C4B—C3B—C2B	119.78 (15)	C12A—C11A—C14A	119.75 (14)
C5A—C4A—C3A	120.18 (16)	C10B—C11B—C12B	118.66 (14)
C5A—C4A—H4A	119.9	C10B—C11B—C14B	121.05 (14)
C3A—C4A—H4A	119.9	C12B—C11B—C14B	120.26 (14)
C5B—C4B—C3B	120.29 (16)	C13A—C12A—C11A	120.19 (14)
C5B—C4B—H4B	119.9	C13A—C12A—H12A	119.9
C3B—C4B—H4B	119.9	C11A—C12A—H12A	119.9
C4A—C5A—C6A	120.81 (15)	C13B—C12B—C11B	120.49 (14)
C4A—C5A—H5A	119.6	C13B—C12B—H12B	119.8
C6A—C5A—H5A	119.6	C11B—C12B—H12B	119.8
C4B—C5B—C6B	121.02 (15)	C12A—C13A—C8A	120.82 (14)
C4B—C5B—H5B	119.5	C12A—C13A—H13A	119.6
C6B—C5B—H5B	119.5	C8A—C13A—H13A	119.6
C7A—C6A—C5A	119.21 (15)	C12B—C13B—C8B	121.09 (14)
C7A—C6A—H6A	120.4	C12B—C13B—H13B	119.5
C5A—C6A—H6A	120.4	C8B—C13B—H13B	119.5
C5B—C6B—C7B	119.19 (16)	N3A—C14A—C11A	179.3 (2)
C5B—C6B—H6B	120.4	N3B—C14B—C11B	179.48 (19)
			
C13A—C8A—N2A—N1A	175.98 (13)	C13B—C8B—N2B—N1B	172.55 (13)
C8A—N2A—N1A—C1A	179.44 (14)	C8B—N2B—N1B—C1B	175.41 (13)
N2A—N1A—C1A—C2A	−179.93 (13)	N2B—N1B—C1B—C2B	177.44 (12)
N1A—C1A—C2A—C3A	0.2 (2)	N1B—C1B—C2B—C3B	−1.4 (2)
C1A—C2A—C3A—O1A	−1.3 (2)	C1B—C2B—C3B—O1B	0.2 (2)

### Hydrogen-bond geometry (Å, º)

**Table:**

*D*—H···*A*	*D*—H	H···*A*	*D*···*A*	*D*—H···*A*
O1*A*—H1*OA*···N1*A*	0.98 (2)	1.77 (2)	2.654 (2)	149 (2)
O1*B*—H1*OB*···N1*B*	0.90 (2)	1.86 (2)	2.653 (2)	147 (2)
N2*A*—H1*NA*···N3*B*^i^	0.91 (2)	2.17 (2)	3.065 (2)	166 (2)
N2*B*—H1*NB*···N3*A*^ii^	0.89 (2)	2.25 (2)	3.098 (2)	158 (2)
C7*A*—H7*A*···O1*B*^iii^	0.93	2.55	3.427 (2)	156
C7*B*—H7*B*···O1*A*^iv^	0.93	2.49	3.413 (2)	172

Symmetry codes: (i) −*x*, −*y*, −*z*; (ii) −*x*+1, −*y*+1, −*z*; (iii) −*x*+1, −*y*, −*z*+1; (iv) −*x*+2, −*y*+1, −*z*+1.
